# Survival trends for patients with retinoblastoma between 2000 and 2018: What has changed?

**DOI:** 10.1002/cam4.5406

**Published:** 2022-12-08

**Authors:** Basel Abdelazeem, Kirellos Said Abbas, Joseph Shehata, Nahla Ahmed El‐Shahat, Mennatullah Mohamed Eltaras, Ibrahim Qaddoumi, Ahmad Samir Alfaar

**Affiliations:** ^1^ McLaren Health Care Flint/Michigan State University Flint Michigan USA; ^2^ Faculty of Medicine Alexandria University Alexandria Egypt; ^3^ Faculty of Medicine Cairo University Cairo Egypt; ^4^ Faculty of Medicine for Girls Al‐Azhar University Cairo Egypt; ^5^ St. Jude Children Research Hospital Memphis Tennessee USA; ^6^ Ophthalmology Department University of Um Ulm Germany; ^7^ Experimental Ophthalmology, Campus Virchow‐Klinikum Charite Universitätsmedizin Berlin Berlin Germany

## Abstract

**Introduction:**

Retinoblastoma (RB) is the most common primary intraocular cancer of childhood. Over the last few decades, a variety of techniques and treatment modalities emerged that improved the survival and ocular salvage rate of patients with RB. We investigated the relative survival trends of patients with RB from 2000 to 2018 by using the Surveillance, Epidemiology, and End Results (SEER) database.

**Design:**

Retrospective database review.

**Methods:**

We extracted data from SEER 18 from 2000 to 2018. All patients with clinically diagnosed RB during the study period were included. We utilized SEER*Stat 8.3.9 and JPSurv software to estimate relative 5‐ and 10‐year survival rates and trends and generated descriptive analyses with IBM SPSS.

**Main Outcome Measures:**

Patient survival rates at 5‐ and 10‐year after RB diagnosis.

**Results:**

RB was diagnosed in 1479 patients within the SEER 18 Program during our study period. The cohort comprised 776 (52.5%) males, 615 (41.6%) non‐Hispanic whites, 487(32.9%) Hispanics, 1030 (69.6%) patients with unilateral disease, and 1087 (73.5%) patients with localized disease. Relative survival trends at 5‐ and 10‐year significantly declined over the study periods (*−0.42%, and −0.50% annually, respectively*) but the decline was not significant in unilateral and bilateral RB cases separately.

**Conclusions:**

Five‐ and ten‐year relative survival trends declined from 2000 to 2018 and were significantly decreasing. Further studies that include more patients are needed to identify the factors contributing to reduced survival of patients with RB over time.

## Graphical abstract

Retinoblastoma was one of the success stories in pediatric oncology, with survival exceeding 98%. However, here we show a decline in the survival of retinoblastoma at the population level. This can be attributed to the new therapies practices in smaller centers with decentralization of services.

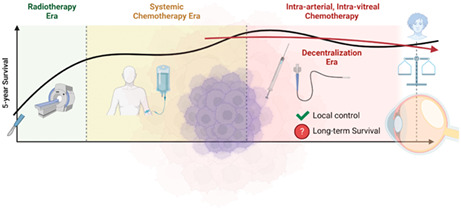

## 1 INTRODUCTION

Retinoblastoma (RB) is the most common malignant retinal cancer of childhood, representing 2.5% to 4% of all childhood malignant tumors,^1^ with an incidence of 1 in 15,000 live births in the United States.^2^ RB can be heritable or nonheritable. Heritable RB is usually caused by germline mutations in the *RB1* tumor suppressor gene,^1^ accounting for 40% of patients with RB. RB primarily occurs as bilateral and/or multifocal disease and at young ages.^3^

Every year, 300 RB cases are expected to be diagnosed in the United States, and 9000 are diagnosed worldwide.^4^, ^5^ Survival is correlated with age at diagnosis, sex, race, the decade of diagnosis, laterality, and economic development.^6^, ^7^, ^8^ Broaddus et al. reported gradually improved survival rates from 92.3% to 96.5% of children with RB from 1975 to 2004 in the United States.^9^ Survival rates in developed countries generally exceed 90%. Consequently, treatment aims have shifted to globe and vision salvage.^5^ However, late diagnosis of advanced‐stage RB is quite common in developing countries.^10^

Many techniques and treatment modalities for managing RB emerged in the twentieth century, including examination of children under anesthesia, brachytherapy, external beam radiation, proton therapy, cryotherapy, and photocoagulation,^11^, ^12^ culminating in standardized systemic chemotherapy protocols with local laser and cryotherapy as front‐line treatments. The early 2000s ushered in ophthalmic artery chemosurgery (OAC) and intravitreal chemotherapy as methods of delivering high concentrations of targeted chemotherapeutics in patients.^13^ Recent studies have investigated gene therapy for the treatment of RB. However, concerns about the effect of new therapies on patient survival remain. To assess the effect of these new therapies and treatment modalities on the current state of RB survival rates in the United States, we performed a retrospective cohort study of relative survival rates from 2000 to 2018 with the Surveillance, Epidemiology, and End Results (SEER) database.

## 2 METHODS

### 2.1 Data source and subject selection

We collected patient data from the SEER 18 database of all RB cases diagnosed in the United States between 2000 and 2018.^14^ We queried the database named “Incidence – SEER Research Data, 18 Registries, Nov 2020 Sub (2000‐2018) – Linked To County Attributes – Time‐Dependent (1990‐2018) Income/Rurality, 1969‐2019 Counties” with SEER*Stat 8.3.9 software. We then extracted data obtained from all patients with RB by using the Site and Morphology International Classification of Childhood Cancer (ICCC) site recode ICD‐O‐3/WHO 2008 version code “V Retinoblastoma.” We included data only from records reporting documented patient ages and malignant tumor behavior in our analysis.^15^ We excluded records that did not include patient survival times or those with data extracted from death certificates and/or autopsy reports. We have extracted the age at diagnosis, sex, race and origin, laterality, stage, sequence, and date and cause of death. Due to the relation of the genetic background of retinoblastoma, we have highlighted the laterality of the disease and its relation to the first year of life. Moreover, we have highlighted the patients that developed further malignancies in the characteristics table.

We merged the stage variables from the SEER stage variables “historic stage A 1973‐2015” and “combined summary stage 2000 (2004‐2017).”. This study adhered to the ethical guidelines of the Declaration of Helsinki and the International Conference on Harmonization. Institutional review board approval and informed consent were not required because of the public nature of the provided data and its consideration as nonhuman subject research. The data were anonymized by the National Cancer Institute before being made publicly available. The study is a retrospective registry‐based cohort study.

### 2.2 Statistical analysis

We used SEER*Stat 8.3.9 software (www.seer.cancer.gov/seerstat)^16^ to estimate relative survival rates at 5 and 10 years after diagnosis. To calculate cancer survival in the absence of other causes of death, we used relative survival as a net indicator.^17^ We then compared the survival rates between patients with unilateral or bilateral RB and calculate the Average Absolute Change in Survival (AAPC) using JPSurv (JoinPoint Survival Model).^18^ We standardized the survival rates for expected survival in the general population by using the survival table “U.S. by SES/geography/race (NHW, NHB, NHAIAN, NHAPI, HISP) 1992‐2016, Ages 0‐99, State‐county (modeled by varied state‐county‐ses).” We analyzed the patient characteristics with IBM SPSS version 27.^19^ Data are presented as frequencies and percentages. We used Tableau software version 2021.2.0 for plotting graphs and calculating trends.^20^ We conducted further survival trend analyses with JPSurv.^18^

## 3 RESULTS

### 3.1 Patient characteristics

We included 1,479 patients from SEER 18 who met our eligibility criteria for analysis (Table 1). Of these patients, 631 (42.7%) were younger than 1 year at RB presentation. Approximately half (*n* = 776, 52.5%) of the patient cohort comprised males, and 615 (41.6%) of patients were white. The majority (*n* = 1030, 69.6%) of patients had unilateral RB. Localized disease occurred in 1,087 (73.5%) patients.

**TABLE 1:** Patients' characteristics

Characteristics		Count	Column *N* (%)
Total		1479	100.0
Age	<01 years	631	42.7
	01 ‐ < 02 years	347	23.5
	02 ‐ < 03 years	286	19.3
	03 ‐ < 04 years	97	6.6
	04 years and more	118	8.0
Sex	Female	703	47.5
	Male	776	52.5
Race and origin	Non‐Hispanic White	615	41.6
	Non‐Hispanic Black	208	14.1
	Non‐Hispanic Asian or Pacific Islander	139	9.4
	Non‐Hispanic American Indian/Alaska Native	16	1.1
	Unknown Race	14	0.9
	Hispanic (All Races)	487	32.9
Laterality	Bilateral	432	29.2
	Unilateral	1030	69.6
	Unknown	17	1.1
Stage	Localized	1087	73.5
	Regional	175	11.8
	Distant	60	4.1
	Unstaged	157	10.6
Sequence	One primary only	1441	97.4
	1st of 2 or more primaries	31	2.1
	2nd of 2 or more primaries	6	0.4
	3rd of 3 or more primaries	1	0.1
Cause of death	Alive	1424	96.3
	Dead from Retinoblastoma	32	2.2
	Dead from other causes	20	1.4
	Dead (missing/unknown COD)	3	0.2

### 3.2 Survival analysis and trends

**FIGURE 1:**
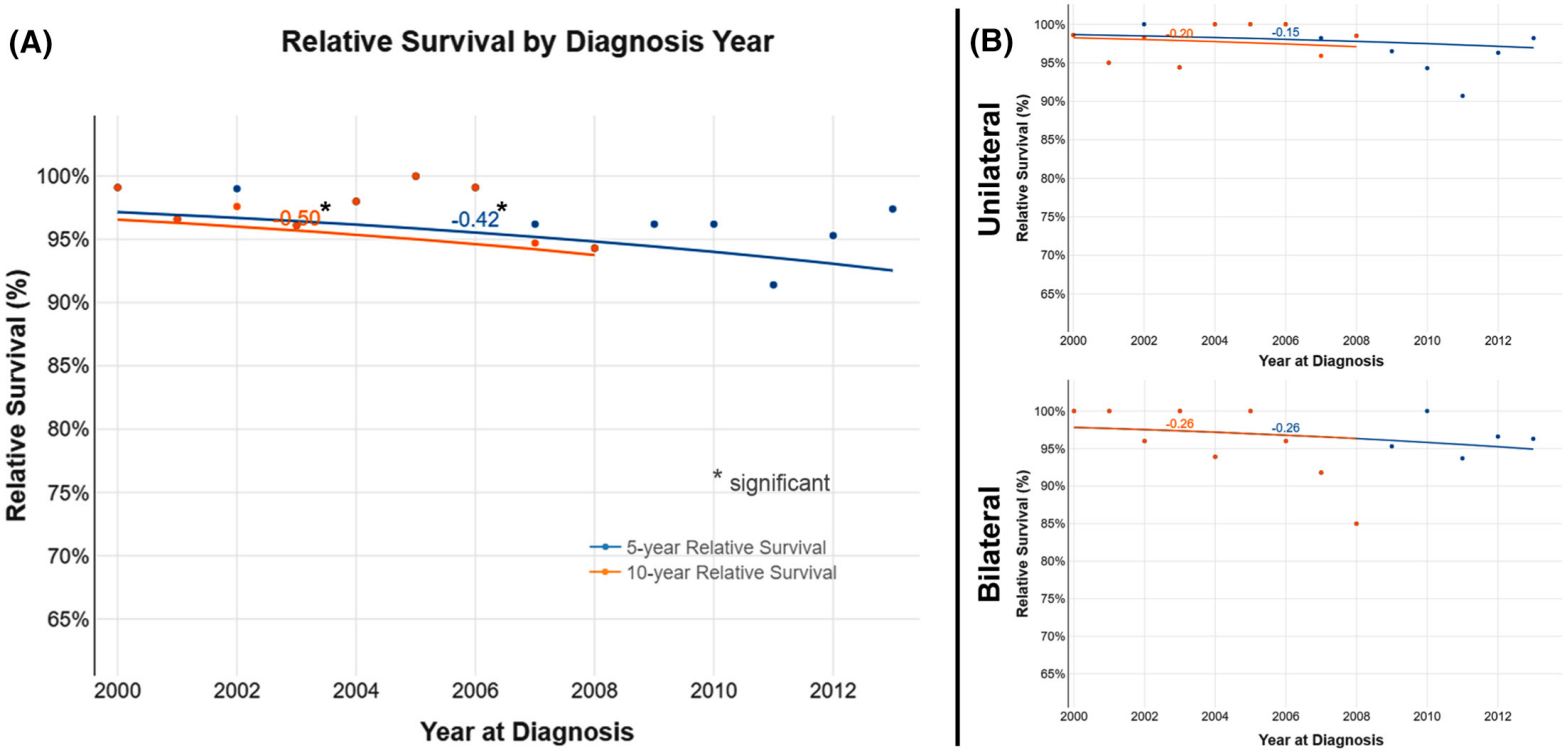
Five‐ and ten‐year relative survival trends among different SEER databases: (A) for All patients, (B) for Unilateral vs. Bilateral Patients.

Figure 1 illustrates the survival trends of patients with RB over the study period. Patient survival declined, irrespective of laterality, from 2000 to 2018. The 5‐year relative survival rate marginally declined (*AAPC = −0.42%, 95% CI* −*0.80 to −0.04*), whereas the 10‐year relative survival rate significantly declined (*AAPC = −0.50%, 95% CI −0.97 to −0.04*). Both the relative and observed causes of death were relatively similar. Both patients with unilateral and bilateral disease (separately) exhibited similar declines in 5‐ and 10‐year relative survival rates. However, this decline was not significant. Therefore, we could not proceed to further analysis of covariates within each subgroup.

## 4 DISCUSSION

The survival rates of patients with RB were initially high, with rates of 99% in the early 2000s, which then fluctuated and declined overall during the study period. It is hard to determine the exact factors contributing to such unexpected decline in survival in a very curable tumor. We speculate that heroic measures (such as intra‐arterial and intra‐vitreal) to salvage eyes even in advanced unilateral RB cases contributed to a such decline in survival. In addition, the decentralization of RB services as smaller centers started treating such rare disease is another contributing factor. The patients volume impacts different outcomes especially when surgical expertise are required.^21^ In rare tumors such as RB, it is even more important.^22^

We believe that when centers with small patients’ volume attempt to use sophisticated salvage therapies further compounds the problem. We urge colleagues to keep saving lives as a priority over saving nonfunctional eyes. Furthermore, there is a risk of causing global decrease in RB survival by disseminating such strategies in LMIC^5^ as many of these countries divert resources from improving pathology services to acquiring such therapies. An even more concerning strategy is being promoted recently called tylectomy that is gaining popularity in China.^23^

The WHO global initiative to improve survival worldwide in six index cancers included RB as one of the cancers.^24^ The ocular oncology community have obligation to support the WHO efforts by focusing on basic needs such as early diagnosis, good enucleation (with long optic nerve stump), and good pathology review and not to jump to advanced technologies before basics.

The survival trends increased from 1975 to 1999, as previously reported.^9^ RB treatment modalities have continued to evolve since the 1950s. Indeed, transitioning from enucleation as the sole treatment for RB to additional treatment techniques such as photocoagulation was first discussed in the 1950s.^11^, ^25^ Cryotherapy was also launched in the 1960s and is effective for treating minor peripheral RB tumors that do not affect vision.^26^ External beam radiation was first used in the 1960s in the United States but introduced severe adverse effects and caused secondary cancers, although it conferred a greater ocular salvage rate.^12^, ^27^ Many facets of RB research led to paradigm shifts in cancer therapy. In 1986, *RB1* was the first cancer gene to be cloned, improving our understanding of the genetic basis of cancer.^28^ Prenatal screening programs for RB were introduced in the early 1990s when methods to detect *RB1* mutations in the fetuses of RB survivor parents were developed.^29^, ^30^, ^31^, ^32^ Moreover, adoption of classification systems, such as the Reese–Ellsworth Classification system for adjusting radiation therapy^33^ and the International Classification of Retinoblastoma to guide chemotherapy,^34^ assisted physicians treating RB during this time period. These developing treatment modalities and other factors contributed to the improved survival rates of patients with RB between 1975 and 1999.

The advent of radiation therapy improved globe salvage rates, except for large RB tumors and vitreal seeds. However, it also increased the risk of radiation‐induced cancers, especially for patients with hereditary disease. Therefore, radiation therapy was replaced with intravenous chemotherapy (popularized in the late 1980s). Properly classifying the disease also improved stratification of patients with expected positive responses to chemotherapy complemented with local consolidative measures, including cryotherapy and laser photocoagulation.^12^, ^34^, ^35^ Systemic chemotherapy, including vincristine, etoposide, and platinum‐based drugs, such as carboplatin, increased the incidence of secondary acute myelogenous leukemia, resulting in higher long‐term mortality rates.^35^, ^36^, ^37^

OAC became popular in the mid‐2000s and provides super‐selective delivery of high concentrations of chemotherapy to RB tumors. Chemotherapy agents that are used commonly for OAC include melphalan, carboplatin, and topotecan. OAC adverse effects were initially higher but are low (<5%) in more recent reports.^12^ The local adverse effects of OAC include vasculopathy, chorioretinal atrophy, delayed vitreous hemorrhage, and blindness secondary to stenosis or occlusion of ophthalmic or retinal arteries. Systemic OAC adverse effects include stroke, iodine allergy, and bone marrow suppression and may have affected the survival trends (both relative and observed) we observed in the mid‐2000s.^38^, ^39^

The risk of secondary cancers is generally high in survivors of heritable diseases, especially those with osteogenic and soft tissue sarcomas, which is not due to long‐term treatment‐related complications or risk of recurrence. Therefore, long‐term follow‐up is required for patients with RB. Bilateral RB is usually diagnosed earlier than unilateral RB because of its hereditary nature. However, we found markedly decreased relative survival of patients with bilateral RB than those with unilateral RB. Patients with bilateral RB have higher rates of other malignant neoplasms, such as pineoblastoma and osteosarcoma.^32^ Moreover, treatment of bilateral RB aims to save at least one eye, increasing the risk of metastases and recurrence and subsequently resulting in high mortality rates, especially with highly selective chemotherapy.^40^, ^41^, ^42^, ^43^ Systemic chemotherapy is recommended to eradicate systemic micro‐metastases because targeted chemotherapy may permit cancer cell escape. Highly selective treatments arose from collaborations between ophthalmology centers and interventional radiology centers to treat patients with RB, but this treatment strategy is less comprehensive than that provided at pediatric cancer centers.

Ultimately, Our suggestion is not to avoid intraarterial or intravitreal chemotherapy but to study the predicting features (clinical, radiologic and genetic) of the risk metastases in the retinoblastoma. Then, to investigate these features in the patients and provide such selective treatments only to the patients who have conducted such investigations. This may lead to new classifications that suit each of the new selective techniques. Moreover, we strongly recommend providing such treatments to the patients that can comply with a strict ophthalmic and pediatric oncology follow‐up. Furthermore, sophisticated technology may be operator‐dependent and not feasible in every center.

Our study has some limitations of note. The SEER database includes patient data from 18 registries, only representing approximately 27% of the US population. In such rare diseases, survival rates are affected by high fluctuations in incidence. Data regarding other medical conditions that may have affected survival rates of patients with RB were not available in the SEER database. Therefore, further research is needed to evaluate survival trends in larger US population of patients with RB. A collaborative registry is needed for long‐term follow‐up of patients with RB who are treated with new therapies. Such registries will permit data collection from all possible sources, including interventional radiology units that treat patients in ophthalmology centers on an outpatient basis, without specifically focusing on highly specialized ocular centers. Furthermore, this will allow studying the effect of various factors on the survival.

## 5 CONCLUSION

We observed a decline in the survival trends of patients with RB from 2000 to 2018. This observation may be attributed to the highly localized therapies used in advanced disease and its subsequent adverse effects. However, additional studies are needed to investigate survival data obtained from active collaborative registries for patients treated with new therapies.

## AUTHOR CONTRIBUTIONS

**Basel Abdelazeem:** Formal analysis (supporting); investigation (supporting); writing – original draft (supporting). **Kirellos Said Abbas:** Formal analysis (supporting); investigation (supporting); writing – original draft (supporting). **Joseph Shehata:** Investigation (supporting); writing – original draft (supporting). **Nahla Ahmed El‐Shahat:** Investigation (supporting); writing – original draft (supporting). **Mennatullah Mohamed Eltaras:** Investigation (supporting); writing – original draft (supporting). **Ibrahim Qaddoumi:** Conceptualization (equal); data curation (supporting); investigation (equal); methodology (equal); project administration (supporting); supervision (supporting); writing – original draft (supporting); writing – review and editing (equal). **Ahmad Samir Alfaar:** Conceptualization (equal); data curation (main); investigation (equal); methodology (equal); project administration (main); supervision (main); writing – original draft (supporting); writing – review and editing (equal).

## FUNDING INFORMATION

We acknowledge the support from the Open Access Publications Funds of the Charite—Universitätsmedizin Berlin.

## CONFLICTS OF INTEREST

The authors declare no conflict of interest.

## Data Availability

Datasets related to this article can be found at www.seer.cancer.gov, an open‐source online data repository hosted by the National Cancer Institute.
